# A Comparison of the Impacts of Different Drying Methods on the Volatile Organic Compounds in Ginseng

**DOI:** 10.3390/molecules29225235

**Published:** 2024-11-05

**Authors:** Yun Xiang, Manshu Zou, Feilin Ou, Lijun Zhu, Yingying Xu, Qingqing Zhou, Chang Lei

**Affiliations:** State Key Laboratory of Chinese Medicine Powder and Medicine Innovation in Hunan (Incubation), Science and Technology Innovation Center, Hunan University of Chinese Medicine, Changsha 410208, China; xiangyun003997@hnucm.edu.cn (Y.X.); zoumanshu@hnucm.edu.cn (M.Z.); fl25800122@126.com (F.O.); 202104030124@stu.hnucm.edu.cn (L.Z.); xuyingying@stu.hnucm.edu.cn (Y.X.); zhouqingqing@stu.hnucm.edu.cn (Q.Z.)

**Keywords:** ginseng, GC-IMS, volatile organic compounds, PCA, Euclidean distance, PLS-DA, CA

## Abstract

Ginseng (*Panax ginseng* C. A. Meyer) is a valuable plant resource which has been used for centuries as both food and traditional Chinese medicine. It is popular in health research and markets globally. Fresh ginseng has a high moisture content and is prone to mold and rot, reducing its nutritional value without proper preservation. Drying treatments are effective for maintaining the beneficial properties of ginseng post-harvest. In this study, we investigated the effects of natural air drying (ND), hot-air drying (HAD), vacuum drying (VD), microwave vacuum drying (MVD), and vacuum freeze drying (VFD) on volatile organic compounds (VOCs) in ginseng. The results showed that the MVD time was the shortest, followed by the VFD, VD, and HAD times, whereas the ND time was the longest, but the VFD is the most beneficial to the appearance and color retention of ginseng. A total of 72 VOCs were obtained and 68 VOCs were identified using the five drying methods based on gas chromatography–ion mobility spectrometry (GC-IMS) technology, including 23 aldehydes, 19 alkenes, 10 alcohols, 10 ketones, 4 esters, 1 furan, and 1 pyrazine, and the ND method was the best for retaining VOCs. GC-IMS fingerprints, principal component analysis (PCA), Euclidean distance analysis, partial least squares discriminant analysis (PLS-DA), and cluster analysis (CA) can distinguish ginseng from different drying methods. A total of 29 VOCs can be used as the main characteristic markers of different drying methods in ginseng. Overall, our findings provide scientific theoretical guidance for optimizing ginseng’s drying methods, aromatic health effects, and flavor quality research.

## 1. Introduction

Ginseng (*Panax ginseng* C. A. Meyer) is a popular natural product often referred to as the king of herbs. Its primary components include ginsenosides, polysaccharides, amino acids, volatile organic compounds (VOCs), and polyacetylene [1], which have beneficial effects, such as enhancing immunity [2], delaying aging [3], antidepressant effects [4], and preventing cardiovascular and cerebrovascular diseases [5]. It has been listed in the “Homology of Medicine and Food” list in the Chinese Ministry of Health Item List since 2014 and has seen widespread application in food, medicine, healthcare, and chemical production.

It is imperative that fresh ginseng be dried promptly after harvesting to prevent mold, bacterial growth, and nutrient loss. Drying is a critical post-harvest operation that retains nutrients, sterilizes, inhibits bacteria, and improves shelf life and storage stability [6]. Traditional and modern methods are used to dry foods and herbs. Traditional methods such as natural air drying (ND), shade drying, and smoke drying have issues related to uncontrolled processes, substandard hygiene, and unreliable quality [7]. Modern technologies include hot-air drying (HAD), microwave vacuum drying (MVD), and vacuum freeze drying (VFD). HAD is the most conventional method due to its low cost and ease of control, but it requires high temperatures and long times, resulting in losses of color, aroma, taste, and nutrients [8]. MVD has a fast speed and uniform heating, but it is costly and energy-intensive [9]. VFD avoids active ingredient destruction and yields low-moisture products, but is costly and has solvent-related limitations [10]. Different drying methods impact aroma-related compounds in foods and herbs. Volatile compounds can be lost during drying, affecting quality and sensory properties [11]. Ginseng has been extensively studied in regard to its ginsenosides and polysaccharides, but the estimation of its VOCs has not received much attention. It is important to determine the quality of herbs based on their odor, which is imparted by VOCs. Pharmacological studies have shown that VOCs have anti-inflammatory, antibacterial, and antitumor effects [12,13,14]. Therefore, ginseng’s quality can be ensured by comparing the effects of different drying methods on its VOCs and screening optimal production methods.

VOCs analysis methods include instrument and sensory analyses [15]. Sensory analysis is often used as an adjunct in VOC detection. The accuracy and reliability of sensory analysis cannot be compared with that of scientific instrumental detection. Instrumental methods include gas chromatography–mass spectrometry (GC-MS), gas chromatography–olfactometry (GC-O), gas chromatography–ion mobility spectrometry (GC-IMS), comprehensive two-dimensional gas chromatography–time-of-flight mass spectrometry (GC×GC-TOF-MS), and electronic nose (E-nose). GC-MS is the most widely used method, but it involves complicated sample pretreatment and long detection times, which is challenging for rapid VOC characterization in traditional Chinese medicine [16,17]. GC-O combines human olfaction with gas chromatography, making it a powerful odor analysis technique [18,19], but lacks reproducibility, stability, and objectivity. GC×GC-TOFMS has strong separation capability, high sensitivity, and automation, but insufficient qualitative ability, slow speed, and low resolution [20]. E-nose is a new aroma detection technology that has high sensitivity, rapid detection, and simple operation [21]; however, it has some disadvantages, such as a limited qualitative and quantitative analysis ability, poor stability, and a significant environmental impact. GC-IMS combines gas chromatography’s high separation capability with ion migration’s fast response, a non-destructive qualitative analysis method using headspace injection for complex target compounds. Compared with other instrumental analysis methods, GC-IMS technology has high efficiency, high sensitivity, high selectivity, no need for sample pretreatment, a short analysis cycle, environmental protection, and a low price. GC-IMS has become an important tool in the field of food flavor and volatile organic compound analysis. The method is widely used in food flavor analysis [22], adulteration identification [23], storage [24], and assessing the influence of food-drying methods [25,26].

Most studies have focused on ginsenosides, total flavonoids, total polysaccharides, and total phenols in ginseng with different drying methods [27,28,29]; however, there has been less research on VOCs. This study aimed to investigate the effects of five drying procedures—namely, ND, HAD, VD, MVD, and VFD—on the appearance and flavor of ginseng. The differences in VOCs in ginseng samples produced using different drying methods were explored using GC-IMS technology combined with principal component analysis (PCA), Euclidean distance analysis, partial least squares discriminant analysis (PLS-DA), and cluster analysis (CA) methods. Our study’s outcomes will help to select the best method for drying ginseng with improved quality, increased production, and enhanced product stability. Additionally, our work provides a valuable reference for large-scale ginseng production.

## 2. Results

### 2.1. Analysis of Visual Characteristics and Color Changes

**Figure 1:**
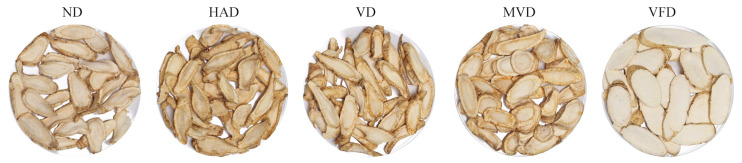
Appearance of ginseng using different drying methods.

The appearance traits of fresh ginseng samples significantly changed after drying. The visual characteristics and color parameters of ginseng after drying using different methods are shown in Figure 1 and Table 1. Using the five drying methods, the color appearance of the ND and MVD samples was considered similar via visual observation, and the sample surfaces were white or light yellow; however, the ND and MVD processing times were the longest and shortest, respectively. The drying times, appearance, and color of HAD and VD were similar, and the surfaces were yellow or brownish-yellow and wrinkled with yellow spots. The appearance of the VFD samples was white and flat, similar to fresh products.

Color is an important indicator to evaluate the quality of food, which affects consumers’ choice of products [30]. Table 1 shows the effects of the different drying methods on the color of ginseng (L, a, b and ΔE). The brightness value of ginseng decreased from 88.06 ± 1.03 to 70.17 ± 4.22. The brightness values of VF-dried samples were the highest among the different drying methods, while that of the HAD sample was the lowest. This may be because the drying and oxidation reduced the brightness caused by the Maillard reaction, while the low-temperature and vacuum environment inhibited the occurrence of enzymatic Browning and Maillard reaction [31,32], thus ensuring ginseng’s original color. The ΔE values were ordered as VFD, MVD, ND, VD, and HAD from high to low, indicating that the VFD method was the best for color retention during the drying process of ginseng. This was related to the degradation of phenols, glycosides, and other substances in the samples exacerbated by the long-term high-temperature environment, thus reducing ginseng’s color.

### 2.2. GC-IMS Analysis of VOCs

**Figure 2:**
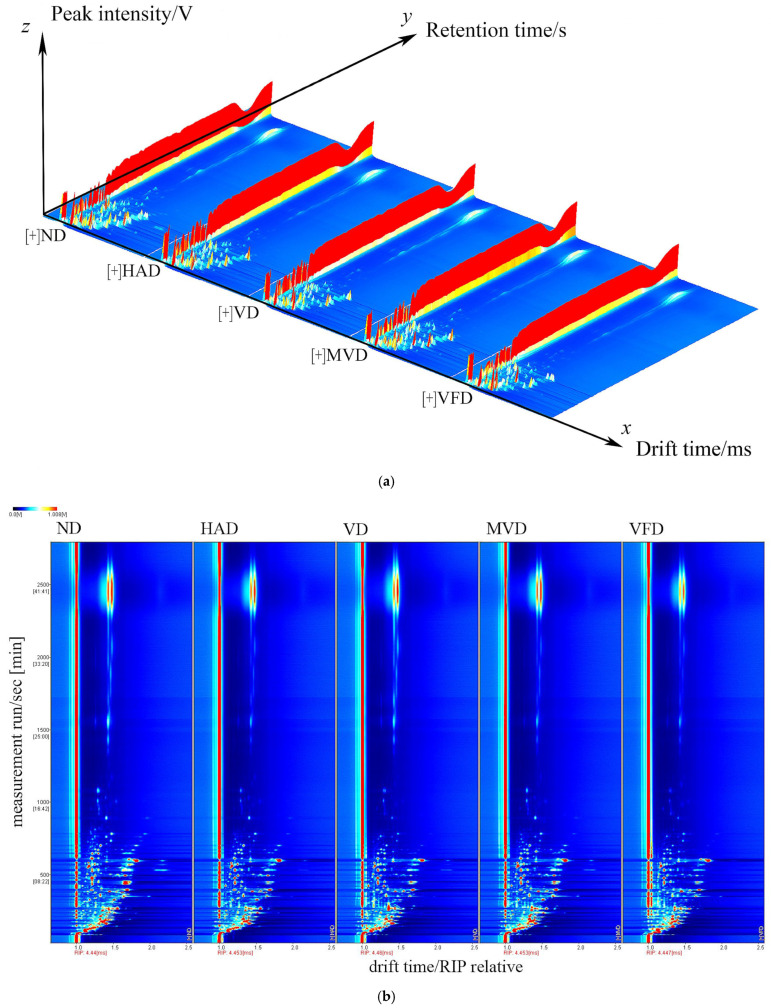

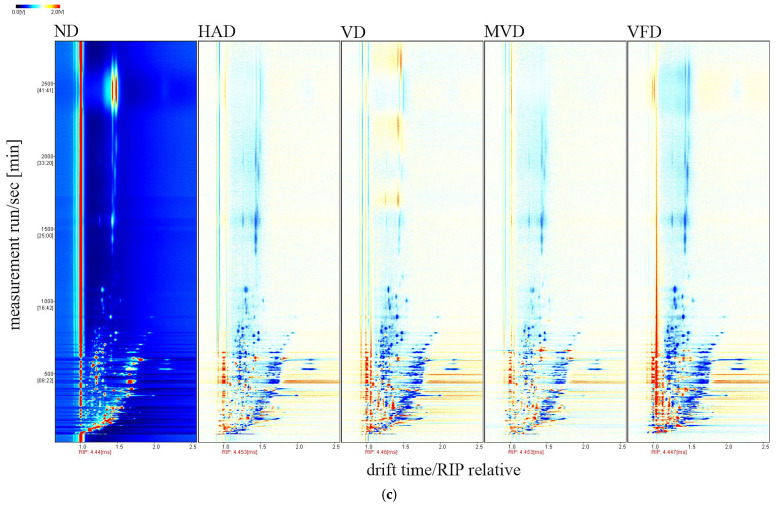
Three-dimensional (**a**) and two-dimensional (**b**,**c**) topographic plots of VOCs in ginseng with different drying methods.

GC-IMS technology was used to identify and analyze the VOCs in ginseng, obtained using a difference comparison model. The horizontal axis represents the ion migration time, the vertical axis represents the gas chromatographic retention time, and the left side indicates the treated reactive ion peak (RIP). Each point on both sides of each RIP represents a volatile organic compound. The color represents the substance’s peak intensity, from blue to red, with darker colors indicating a greater peak intensity [33]. In Figure 2, the three axes represent the migration time (*X*-axis), retention time (*Y*-axis), and signal peak intensity (*Z*-axis), respectively. The differences in VOCs between the different samples can be visually seen in Figure 2a. A top view is depicted in Figure 2b for ease of observation. The differences in the VOCs between the five samples are evident in the figure. The ND spectra samples were selected as a reference to further visually compare the VOCs differences, which were subtracted from the other sample spectra to obtain the difference comparison diagram for each sample, as shown in Figure 2c. The subtracted background is white if the target sample and reference have the same VOCs content; red means the target sample has a higher concentration of the substance than the reference; and blue means the target sample has a lower concentration than the reference. The analysis showed that the components represented by red dots were present in all five samples in varying amounts. The red dots were most abundant in ND, suggesting that this drying method was beneficial for preserving the VOCs in ginseng.

### 2.3. Identification of VOCs in Different Samples

Although the two-dimensional spectrum of GC-IMS could intuitively show the contents and differences in VOCs in ginseng obtained using the different drying methods, it could not accurately judge the individual substances in the samples. GC retention index (NIST 2020) and IMS migration time databases were used to characterize the VOCs in the ginseng samples after using the different drying methods, according to their relative migration time and gas chromatography retention index. The qualitative results and odor descriptions of the VOCs in ginseng after using the different drying methods are shown in Table 2. The odor of VOCs described mainly refer to the database definitions, including https://www.flavornet.org (accessed on 10 October 2004), https://www.femaflavor.org (accessed on 10 October 2024), and https://www.chemicalbook.com (accessed on 10 October 2024). According to the different retention indices and migration times of the characteristic peaks of each substance, 72 signal peaks were determined, and 68 types of VOCs were qualitatively detected, including 23 aldehydes, 19 alkenes, 10 alcohols, 10 ketones, 4 esters, 1 furan, and 1 pyrazine. Some compounds showed multiple signals in the ion migration spectrum during detection, which was due to some components exhibiting dimers and monomers simultaneously. The monomers and dimers were the same substance. However, protonated molecules in the ionization region were promoted to combine with neutral molecules to form dimers due to the high concentration of these compounds. As a result, the reactants’ protons were transferred to these high-proton-affinity compounds, and dimers or polymers were then formed [34].

### 2.4. Comparison of the Fingerprints of VOCs

**Figure 3:**
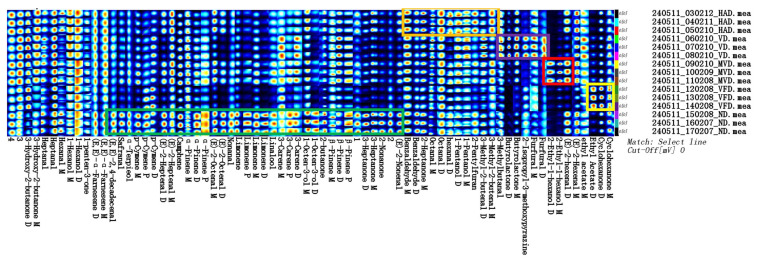
The VOCs fingerprints of ginseng with different drying methods.

A fingerprint can show the intensity of VOCs spots in different samples for analysis and comparison, enabling the determination of changes in individual substances [35]. Figure 3 represents the VOCs fingerprints in the ginseng samples produced using the different drying methods. Each row in the fingerprint represents all signal peaks selected from a ginseng sample; each column represents the signal peaks of the same VOCs in different ginseng samples; each unit represents the content of substances at different times; and the color represents the VOCs contents. The brighter the color, the higher the content. The unknown but different components are numbered 1–4. The green box indicates high VOCs contents in ND, the orange box indicates high VOCs contents in HAD, the purple box indicates high VOCs contents in VD, the red box indicates high VOCs contents in MVD, and the yellow box indicates high VOCs contents in VFD. The results show differences in the VOCs compositions using the different drying methods. The ND samples had the reddest bright spots, the most abundant types of VOCs, and the highest contents of some components. The MVD and VFD samples contained few red bright spots, the lowest number of VOCs, and low contents of most components. In Figure 3, the green box represents the characteristic VOCs using ND, which mainly included (E, E)-2,4-decadecenal, safranal, α-terpieol, p-cymene, (E)-2-heptenal, camphene, (E)-2-octenal, α-pinene, β-pinene, nonanal, limonene, linalool, 3-carene, 1-octen-3-ol, 2-butanone, 3-heptanone, 2-nonanone, and (E)-2-nonenal. The orange box represents the characteristic VOCs using HAD, which mainly included 2-heptanone, benzaldehyde, nonanal, hexanal, 1-pentanol, octanal, and 3-methyl-2-butenal. The purple box represents the characteristic VOCs using VD, which mainly included butyrolactone,3-methylbutanal, 2-isopropyl-3-methoxypyrazine, and furfural. The red box represents the characteristic VOCs using MVD, which mainly included 2-ethyl-1-hexanol and (E)-2-hexenal. The yellow box represents the characteristic VOCs using VFD, which mainly included cyclohexanone and ethyl acetate. Therefore, the VOCs contents can serve as an important index for distinguishing between different ginseng-drying methods.

### 2.5. Chemometric Analysis

#### 2.5.1. Principal Component Analysis (PCA)

**Figure 4:**
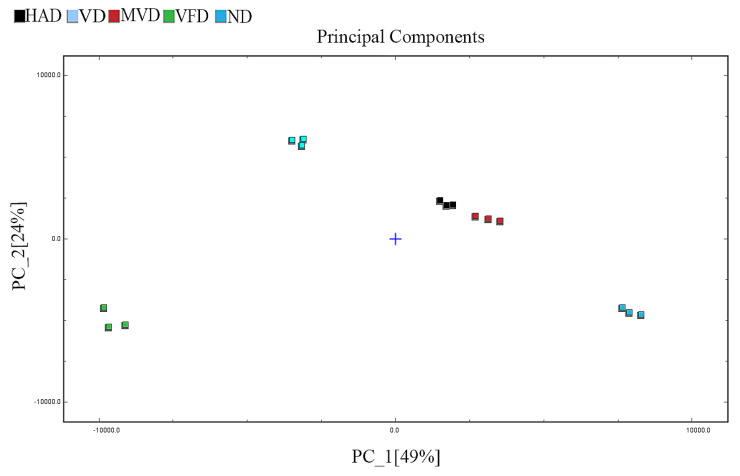
PCA score plot.

**Figure 5:**
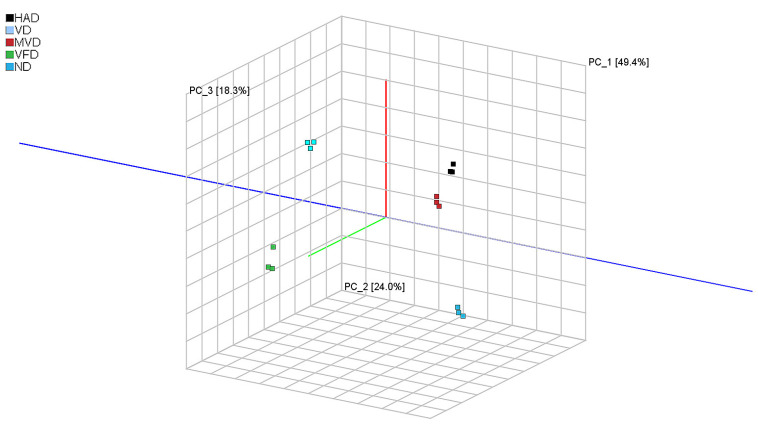
PCA 3D scatter plot.

PCA is a multivariate statistical method used to test the correlations between multiple variables, primarily through assessing the signal strength of volatile substances in a substance to highlight differences between samples. The Dynamic PCA plug-in in VOCal data processing software (from G.A.S., Dortmund, Germany, version 2.0.0) was used to analyze the principal components of VOCs in ND, HAD, VD, MVD, and VFD samples, and the results are shown in Figure 4. The figure shows that the first two principal components explained 73% of the total variance among the samples from the five different drying methods (PC1, 49.0%; PC2, 24.0%). It can be clearly seen from the figure that the distance between HAD and MVD is small, indicating that the VOCs were relatively similar. However, the large distances between other samples indicate that there are certain differences in the VOCs content of ginseng after different drying methods. These differences were also visualized in the 3D spatial scatter diagram (Figure 5), in which the first three principal compounds accounted for 91.7% of the total variance. The original variables contained the 3D information of the retention time (PC1), drift time (PC2), and ion signal strength (PC3) (as variables, mainly as observations) for comprehensive analysis and PCA. Therefore, the results revealed that GC-IMS coupled with PCA can rapidly distinguish ginseng undergoing different drying methods.

#### 2.5.2. Euclidean Distance Analysis

**Figure 6:**
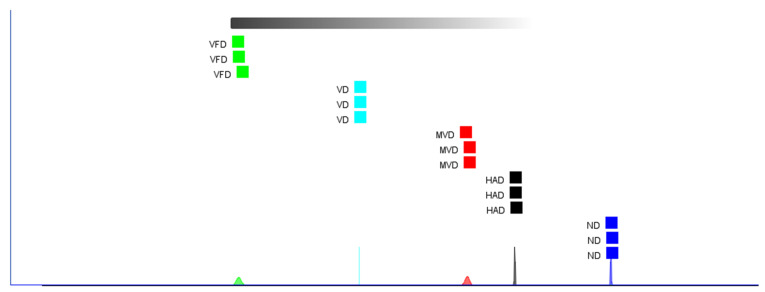
Fingerprint similarity based on Euclidean distance of different samples.

Additionally, we used Euclidean distance, a cluster analysis method that refers to the real distance between two points, reflecting the closeness of the study subjects [36]. If the distance was large, the difference between the two was also large and showed a positive correlation. The Euclidean distance of the ginseng samples treated with the different drying methods is shown in Figure 6. The results of the Euclidean distance analysis show that the distances between the ginseng samples of different origins were clearly distinguished. The distance between MVD and HAD was relatively close, indicating little difference between them. Meanwhile, the distance between VFD and ND was the furthest, indicating a big difference between them. This is similar to the PCA results.

#### 2.5.3. Partial Least Square-Discriminant Analysis (PLS-DA)

**Figure 7:**
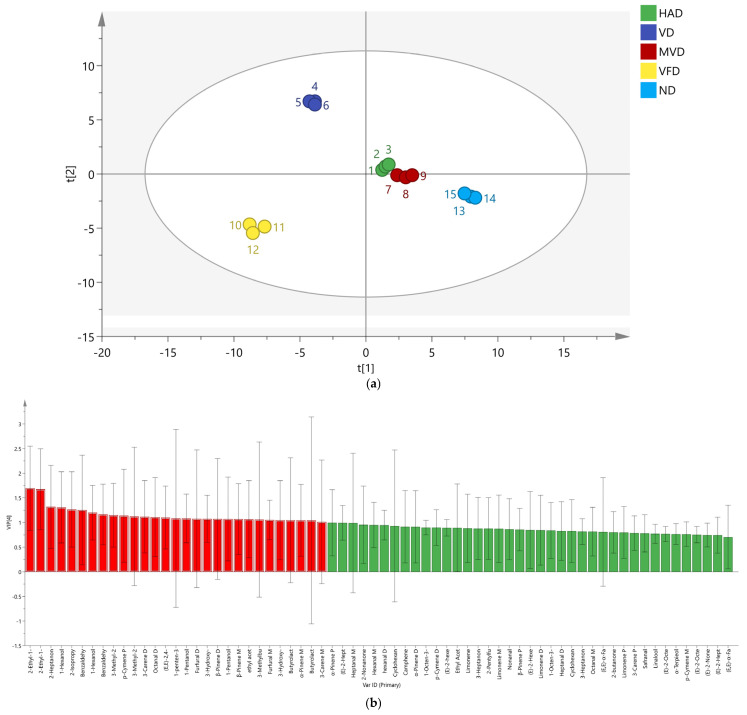

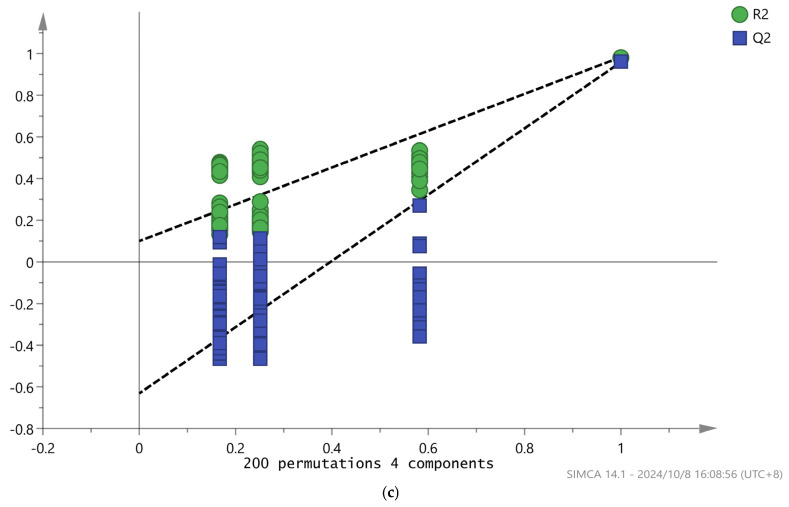
The PLS-DA score plots of region multi-classification (**a**); VIP diagram of the PLS-DA model (**b**); the permutation test results (*n* = 200) of the PLS-DA model (**c**).

PLS-DA is a supervised approach that can identify hidden variables that compromise the robustness of the model and highlight differences between groups [33]. The peak volume results of 68 VOCs from five ginseng samples were normalized and PLS-DA scores were obtained. The results are shown in Figure 7a; R^2^X = 0.981, R^2^Y = 0.993, Q^2^ = 0.983, and are all greater than 0.5, indicating that the established model is stable and reliable. The distance between HAD and MVD was the smallest, and there was a significant gap between the other three groups, consistent with the PCA.

The score chart shows that the supervised analysis method can better distinguish the five ginseng samples, and the variable important in projection (VIP) value can be obtained. VIP values are often used to reflect the importance of PLS-DA model variables, where VIP is greater than 1, indicating a high contribution to the overall discriminant model [33]. As shown in Figure 7b, there are 29 characteristic VOCs with a VIP greater than 1 (indicated by the red bar chart), including 2-Ethyl-1-hexanol D, 2-Ethyl-1-hexanol M, 2-Heptanone M, 1-Hexanol D, 2-Isopropyl-3-methoxypyrazine, Benzaldehyde M, 1-Hexanol M, Benzaldehyde D, 3-Methyl-2-butenal M, p-Cymene P, 3-Methyl-2-butenal D, 3-Carene D, Octanal D, (E,E)-2,4-decadecenal, 1-penten-3-one, 1-Pentanol M, Furfural D, 3-Hydroxy-2-butanone M, β-Pinene D, 1-Pentanol D, β-Pinene M, ethyl acetate M, 3-Methylbutanal, Furfural M, 3-Hydroxy-2-butanone D, Butyrolactone M, α-Pinene M, Butyrolactone D, and 3-Carene M, indicating that they play an important differentiating role in the five ginseng drying methods. To assess model overfitting, we conducted 200 cross-validations of R^2^ and Q^2^. The results are shown in Figure 7c, R^2^ = 0.0998, Q^2^ = −0.631, indicating that the model was proven to be reliable without overfitting and could be used to describe the differences in VOCs in ginseng using five different drying methods.

#### 2.5.4. Cluster Analysis (CA)

**Figure 8:**
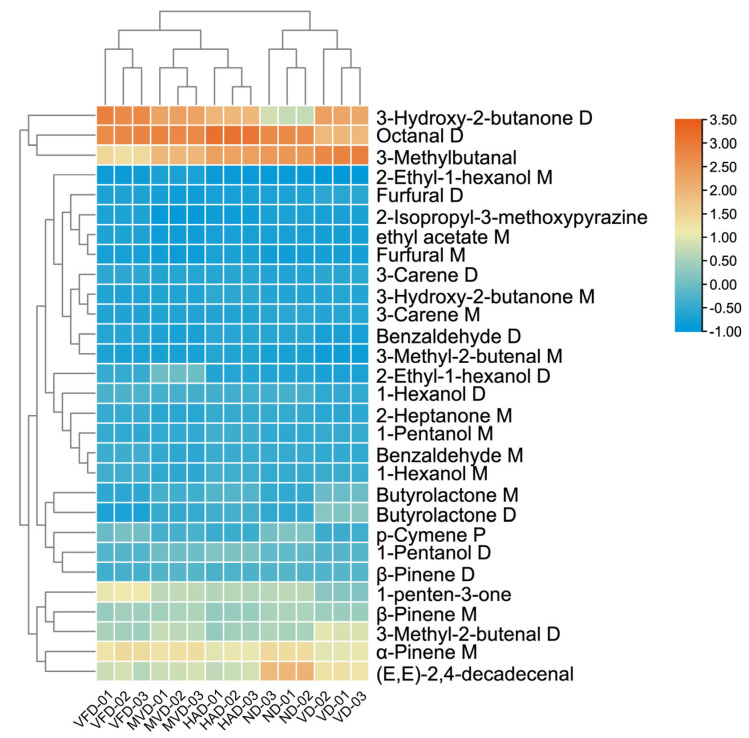
CA of characteristic VOCs in ginseng with different drying methods.

In order to further elucidate whether the 29 components can serve as characteristic VOCs to distinguish and identify the five drying methods of ginseng, clustering heat maps are generated based on their peak intensity values. As shown in Figure 8, the horizontal coordinate represents different samples, and the vertical coordinate represents VOCs. Orange indicates an increase in the relative peak intensity value, while blue indicates a decrease. The 29 VOCs can effectively distinguish and identify the five drying methods of ginseng, and visually show the relative content of components among different drying methods. The markers of MVD are not obviously defined by VOCs, and the contents of 1-penten-3-one, Octanal D, (E, E)-2,4-decadecenal, and 3-Methylbutanal are relatively high in VFD, HAD, ND, and VD, respectively. In addition, based on the heat maps combined with CA, we can distinguish the VOCs’ differences in the five drying methods in ginseng, which are similar to the results of PCA, PLS-DA, and Euclidean distance. The CA roughly categorizes the five sample groups into three categories, VFD, MVD and HAD, and ND and VD, with good reproducibility within each group.

## 3. Discussion

In this study, GC-IMS technology was applied to analyze the VOCs in ginseng using different drying methods for the first time. Fresh ginseng was treated using ND, HAD, VD, MVD, and VFD. The color and appearance of the VFD-treated ginseng samples were most similar to those of fresh ginseng via visual observation. The ginseng samples after treatment with MVD and ND were white or light yellow, while the ginseng samples with HAD and VD treatment were yellowish or brown-yellow and wrinkled. In terms of color, the L (88.06 ± 1.03) of the VFD samples was the highest, and the ΔE (11.94 ± 1.41) was the lowest, indicating that the dry products’ brightness after VFD treatment was the highest and the Browning was the smallest. Moreover, the ΔE (26.52 ± 3.23) after HAD treatment was the largest, indicating that this method was not conducive to ginseng color retention. According to these results, the appearance of the ginseng samples was greatly influenced by the environment’s temperature and pressure during drying. This is due to the rapid evaporation of surface water during drying, resulting in significant structural and shape changes. Furthermore, the long-term high-temperature environment increases the probability of sample oxidation and Maillard reactions. Similar results have also been reported by Ning [37] for red ginseng. In contrast, ND uses sunlight and circulating air to evaporate water in traditional Chinese medicine more gently than heating and drying, but takes longer. The ΔE (20.05 ± 1.73) of MVD was low, which may have been due to the inactivation of oxidase and peroxidase in the samples caused by electromagnetic waves, thus reducing the occurrence of enzymatic Browning reaction [38]. VFD at lower temperatures resulted in ice crystals in vacuum freeze-dried samples. After sublimation, the samples largely maintained their original structure and shape. In summary, VDF is the most beneficial to the appearance and color retention of ginseng in the drying process, followed by MVD and ND.

However, sensory evaluation is subjective, so GC-IMS technology was used to objectively analyze the ginseng samples’ odor and compare the different drying methods’ effects on the ginseng’s VOCs. A total of 72 VOCs were obtained and 68 VOCs were identified in ginseng samples using GC-IMS. The identified VOCs included 23 aldehydes, (33.8%), 19 alkenes (27.9%), 10 alcohols (14.7%) and 10 ketones (14.7%), 4 esters (5.9%), 1 furan (1.5%) and 1 pyrazine (1.5%). Through a comparative fingerprint analysis of VOCs using the five different drying methods, it was found that the types of VOCs were basically the same, but the contents of VOCs were quite different. The VOCs contents in ginseng dried using ND were higher and its odor was best preserved. Most aldehydes, alkenes, ketones, and alcohols had high content in this method. However, this method was susceptible to natural conditions and was time-consuming. These problems would be noteworthy in large-scale production processes. The VOCs contents in ginseng dried using HAD, VD, and MVD were reduced and differed less. This phenomenon may have resulted from the ambient temperature during heating and drying, which can significantly influence the VOCs’ matter content in ginseng samples. As a food and Chinese herbal medicine, ginseng inevitably contains numerous VOCs with poor stability. These compounds are decomposed by heat after temperature rise, which may also be one of the reasons why the VOCs’ matter content in ginseng after heating and drying is relatively similar. The VOCs content in ginseng dried using VFD also decreased, and the difference was significant compared to other drying methods. This may have been due to the low temperature inhibiting the activity of the synthase enzyme involved in ginseng VOCs production. In general, compounds with poor stability in food and Chinese medicine are easily destroyed by heat. Compared with drying methods such as high temperature heating, VFD can better retain active ingredients and thus maintain the efficacy of Chinese medicinal materials. However, in this study, it was found that VFD has a greater impact on the VOCs of ginseng, resulting in odor loss. Similar results were also reported in the related literature [39,40]. The VOCs in VFD are mainly ethyl acetate and other esters, which have a fruity odor and are often used in the preparation of fruity and wine flavors.

The results of PCA, Euclidean distance analysis, PLS-DA analysis, and CA showed that the five drying methods in ginseng were well-separated and could be distinctly distinguished. In general, the types and contents of VOCs in MVD and HAD had been similar and close. Moreover, based on the importance of variables in the PLS-DA model, the contribution of each variable to the classification is quantified. If the VIP value is greater than 1, the compound can be used as potential characteristic markers. The higher the VIP value, the greater the difference in VOCs between different dried ginseng samples. A total of 29 VOCs can serve as primary characteristic markers to distinguish different drying methods in ginseng, including 2-Ethyl-1-hexanol M, 2-Ethyl-1-hexanol D, 2-Heptanone M, 1-Hexanol D, 2-Isopropyl-3-methoxypyrazine, Benzaldehyde M, and others. HCA confirmed that these VOCs could be used as the signature substances of five drying methods in ginseng. 1-penten-3-one is a major characteristic VOCs in VFD, with strong pungent odors, and the ability to induce the production of reactive oxygen species (ROS) and activate downstream defense responses in plants [41]. Octanal D can be used as a main characteristic VOC in HAD, and it possesses aldehyde, waxy, citrus, orange, fruity, and fatty odors. It is commonly used to produce a sweet orange aroma in the food industry. (E, E)-2,4-decadecenal is a major characteristic VOCs in ND, with cucumber, melon, citrus, pumpkin, and nutty odors. 3-Methylbutanal is a key characteristic VOCs in VD, with chocolate and fat odors, and is mainly used for food flavoring.

In this study, the GC-IMS rapid analysis method for the VOCs in ginseng using different drying methods was established. Compared with HS-SPME/GC-MS [42] analysis results, more compounds were detected in this study, the samples did not require pretreatment, and the analysis cycle was shorter. Therefore, GC-IMS technology has significant advantages in the analysis of VOCs. Furthermore, ND was determined to be the most suitable for retaining the VOCs and odor of ginseng, based on a comprehensive comparison of the effects of the different drying methods on the ginseng’s VOC types, content, and appearance. This study provides scientific theoretical guidance for optimizing ginseng-drying methods and studying flavor quality.

## 4. Materials and Methods

### 4.1. Samples

Ginseng was collected from Songjiang Town, Fusong County, Baishan City, Jilin Province, China; was transported to the laboratory within 24 h of harvest using a cold chain; and was frozen at −40 °C until the drying experiments.

### 4.2. Drying Procedures

The ginseng was washed with clean water, sliced, and then randomly divided into groups for the different drying methods, including natural air drying (ND), hot-air drying (HAD), vacuum drying (VD), microwave vacuum drying (MVD), and vacuum freeze drying (VFD). All freshly harvested ginseng samples were dried to a constant weight. For ND, the samples were evenly spread on stainless steel trays and exposed to natural sunlight for 14 h. For HAD, a hot-air oven (ZXRD-A7140, Zhicheng Analytical Instrument Manufacturing Co., Ltd., Shanghai, China) was heated to 60 °C, and the samples were then placed in the oven and dried at 60 °C for 11 h. For VD, the samples were treated using a vacuum oven (BZF-50, Boxun Medical Biological Instrument Co., Ltd., Shanghai, China) at −0.09 MPa and 60 °C for 10 h. For VFD, the samples were pre-frozen at −80 °C and then dried using a freeze-dryer (Alpha 2-4 LSCbasic, CHRIST, Hagen, Germany) for 7 h, where the condenser temperature and chamber pressure were −80 °C and −0.1 MPa, respectively. For MVD, the samples were placed in a microwave oven (WB-5, Famouk Co., Ltd., Fuzhou, China) and dried at 560 W for 1.5 h. The temperature and pressure were maintained at 60 °C and −0.08 MPa, respectively. Each drying process was repeated three times.

Portions of the ginseng samples prepared using the different drying methods were pulverized with a universal high-speed pulverizer (DFT-100A, Lin Da Machinery Co., Ltd., Wenling, China), sieved through a 60-mesh sieve, packed in closed plastic bags, and stored at −40 °C for VOC analysis.

### 4.3. Color Measurement

The color parameters (*L*, *a*, and *b* values) of the dried samples were quantitatively determined using a fully automatic colorimetric analyzer (Color Quest XE, Hunter Lab, Boston, MA, USA). The color difference (ΔE) was calculated using Equation (1) [43].

(1) $$\Delta E=\sqrt{{\left(L^{*}-L\right)}^{2}+{\left(b^{*}-b\right)}^{2}+{\left(a^{*}-a\right)}^{2}}$$

where *L*, *a*, and *b* are the brightness, red–green, and blue–yellow values. L* = 91.75, a* = −1.73, and b* = 3.52 were the color values of the standard white plate.

### 4.4. GC-IMS Analysis

#### 4.4.1. Headspace Sampling Conditions

Ginseng samples of 0.5 g were accurately weighed and placed in a 20 mL headspace bottle. The incubation temperature was 70 °C and 500 rpm for 20 min in a shaker. The injection volume was 500 µL, and the injection needle temperature was 85 °C. Three parallel groups were determined for each sample.

#### 4.4.2. GC-IMS Conditions

The temperature of the MXT-5 capillary chromatography column (15 m × 0.53 mm × 1 μm, Restek Company of the United States, Bellefonte, PA, USA) and IMS were 60 °C and 45 °C, respectively. The ionization source was tritium (^3^H), the migration tube length was 53 mm, and the electric field strength was 500 V/cm. High-purity nitrogen (purity ≥ 99.999%) was used as the carrier and drift gas. The flow rate of the drift gas was set at 75 mL/min. The programmed flow rate of the whole phase was as follows: the initial flow rate of 2.00 mL/min was maintained for 2 min, linearly increased to 10.00 mL/min within 8 min, then linearly increased to 100.00 mL/min within 10 min, and maintained for 39 min. The total analysis time was 59 min, and the inlet temperature was 85 °C.

#### 4.4.3. Statistical Analysis

The mixing of 6 ketones (2-butanone, 2-pentanone, 2-hexanone, 2-heptanone, 2-octanone and 2-nonone) was tested to establish a calibration curve of the retention time and retention index. Then, the retention index of the target substance was calculated from the retention time. A qualitative analysis of the target was performed using the built-in GC Retention Index (NIST 2020) database and IMS migration time database in the VOCal data-processing software.

The VOCal plug-in data-processing software, such as Reporter (Version 11.x), Gallery Plot (Version 1.1.0.2), and Dynamic PCA (version 0.0.3) were used to generate the three-dimensional spectrum, two-dimensional spectrum, difference spectrum, fingerprint, PCA, and Euclidean distance diagram of the VOCs for comparison between the samples. The CA was performed by TBtools software (version v2.119). The PLS-DA VIPs were calculated using the SIMCA software (version 14.1).

## 5. Conclusions

In summary, this study established a GC-IMS rapid analysis method for ginseng VOCs using different drying methods. A total of 72 VOCs were obtained and 68 VOCs were identified using the five drying methods, including 23 aldehydes, 19 alkenes, 10 alcohols, 10 ketones, 4 esters, 1 furan, and 1 pyrazine. Among them, aldehydes and alkene compounds were the primary components of the VOCs in ginseng. In addition, the effects of five different drying methods (ND, HAD, VD, MVD, and VFD) on ginseng’s appearance characteristics and VOCs were evaluated. The results showed that VFD produced a better appearance than other drying methods, while ND was the best drying method for ginseng in order to retain its VOCs and odor. Based on statistical analysis, including PCA, Euclidean distance, CA, and PLS-DA, the similarities and differences between the five different drying methods were found. A total of 29 VOCs can be used as key factors for the identification and differentiation of drying methods, and it was proved that these VOCs could be used to distinguish the five drying methods in ginseng. After a comprehensive comparison, it was concluded that the ND method was better than the other four drying methods. Nonetheless, its drying time was longer, and it was affected by external factors.

Furthermore, this study has limitations: the experiment did not investigate the effect of different drying temperatures on ginseng using the same drying method. There is no systematic study on the effects of different drying methods on the microstructure, physical properties (such as drying time, rehydration ratio, and shrinkage rate), and non-volatile components of ginseng. Future research should explore a wider range of the drying conditions, ginseng varieties, and analytical methods to fully understand and optimize the drying processing techniques of ginseng varieties.

This study provides new ideas and directions for the drying and processing technology of ginseng production. However, when selecting the drying method, we need to comprehensively consider the material characteristics, production conditions, economic factors, environmental impacts, and safety aspects to ensure the efficiency, economy, and safety of the ginseng-drying process.

## Figures and Tables

**Table 1:** The color parameters of ginseng using different drying methods.

Drying	*L*	*a*	*b*	Δ*E*
ND	72.72 ± 6.89 ^c^	1.68 ± 0.76 ^bc^	15.91 ± 1.76 ^bc^	23.32 ± 5.66 ^ab^
HAD	70.17 ± 4.22 ^c^	2.64 ± 0.70 ^a^	18.01 ± 1.61 ^a^	26.52 ± 3.23 ^a^
VD	70.44 ± 2.04 ^c^	1.89 ± 0.31 ^b^	17.87 ± 0.34 ^a^	25.98 ± 1.69 ^a^
MVD	77.68 ± 1.96 ^b^	1.18 ± 0.43 ^c^	17.41 ± 1.45 ^ab^	20.05 ± 1.73 ^b^
VFD	88.06 ± 1.03 ^a^	−0.91 ± 0.2 ^d^	14.82 ± 1.27 ^c^	11.94 ± 1.41 ^c^

where *L*, *a*, *b*, Δ*E* are the brightness, red–green and blue–yellow values, and color difference. Different numbers in the same column with different letters as superscript were significantly (*p* < 0.05) different.

**Table 2:** VOCs in ginseng with different drying methods by GC-IMS.

No	Compounds	CAS	Molecular Formula	RI	Rt/s	Dt/ms	Odor Description
1	(*E*, *E*)-*α*-Farnesene D	C502614	C_15_H_24_	1487.1	2464.654	1.48579	citrus, herbal, lavender, neroli
2	(*E*, *E*)-*α*-Farnesene M	C502614	C_15_H_24_	1485.7	2454.604	1.42258	citrus, herbal, lavender, green
3	Safranal	C116267	C_10_H_14_O	1208.9	1077.33	1.28714	herbal, phenolic, tobacco, spicy
4	*α*-Terpieol	C10482561	C_10_H_14_O	1188.8	1014.845	1.28968	floral, lilac, terpenic
5	Nonanal	C124196	C_9_H_18_O	1104.8	790.476	1.49418	rose, citrus, strong oily
6	2-Isopropyl-3-methoxypyrazine	C25773404	C_8_H_12_N_2_O	1095.1	768.11	1.25062	mung bean
7	Linalool	C78706	C_10_H_18_O	1086.2	748.099	1.21862	citrus, rose, woody, blueberry
8	(*E*)-2-Octenal M	C2548870	C_8_H_14_O	1065.5	703.366	1.32707	fresh cucumber, fatty, green herbal, banana, green leaf
9	(*E*)-2-Octenal D	C2548870	C_8_H_14_O	1065.5	703.366	1.82308	fresh cucumber, fatty, green herbal, banana, green leaf
10	*p*-Cymene M	C99876	C_10_H_14_	1020.4	615.079	1.21506	fresh, citrus, terpene, woody, spice
11	*p*-Cymene D	C99876	C_10_H_14_	1021.1	616.256	1.31284	resh, citrus, terpene, woody, spice
12	*p*-Cymene P	C99876	C_10_H_14_	1017.8	610.371	1.7253	fresh, citrus, terpene, woody, spice
13	Octanal D	C124130	C_8_H_16_O	1013.9	603.308	1.81241	aldehyde, waxy, citrus, orange, fruity, fatty
14	Octanal M	C124130	C_8_H_16_O	1008	592.713	1.41418	aldehyde, waxy, citrus, orange, fruity, fatty
15	1-Octen-3-ol D	C3391864	C_8_H_16_O	983.8	545.935	1.59477	mushroom, lavender, rose, hay
16	Benzaldehyde M	C100527	C_7_H_6_O	961.9	502.347	1.1525	bitter almond, cherry, nutty
17	Benzaldehyde D	C100527	C_7_H_6_O	961.9	502.347	1.46539	bitter almond, cherry, nutty
18	(*E*)-2-Heptenal D	C18829555	C_7_H_12_O	959.7	498.128	1.6621	spicy, green vegetables, fresh, fatty
19	(*E*)-2-Heptenal M	C18829555	C_7_H_12_O	957.8	494.613	1.25811	spicy, green vegetables, fresh, fatty
20	Heptanal D	C111717	C_7_H_14_O	899.9	396.891	1.69906	fresh, aldehyde, fatty, green herbs, wine, fruity
21	Heptanal M	C111717	C_7_H_14_O	902.7	401.109	1.35053	fresh, aldehyde, fatty, green herbs, wine, fruity
22	Cyclohexanone D	C108941	C_6_H_10_O	893.3	387.049	1.44426	strong pungent, earthy
23	Cyclohexanone M	C108941	C_6_H_10_O	892.3	385.643	1.15646	strong pungent, earthy
24	1-Hexanol M	C111273	C_6_H_14_O	869.7	354.709	1.32808	fresh, fruity, wine, sweet, green
25	1-Hexanol D	C111273	C_6_H_14_O	867.2	351.447	1.64167	fresh, fruity, wine, sweet, green
26	(*E*)-2-Hexenal M	C6728263	C_6_H_10_O	853.9	334.544	1.17881	green, banana, fat
27	(*E*)-2-hexenal D	C6728263	C_6_H_10_O	849.1	328.569	1.51598	green, banana, fat
28	hexanal D	C66251	C_6_H_12_O	797.3	271.21	1.55279	fresh, green, fat, fruity
29	Hexanal M	C66251	C_6_H_12_O	800.8	274.795	1.26863	fresh, green, fat, fruity
30	1-Pentanol D	C71410	C_5_H_12_O	767.1	241.335	1.52334	balsamic
31	1-Pentanol M	C71410	C_5_H_12_O	768.4	242.53	1.24801	balsamic
32	3-Methylbutanal	C590863	C_5_H_10_O	668.3	164.979	1.40801	chocolate, fat
33	Ethyl Acetate D	C141786	C4H8O_2_	628.6	144.91	1.33439	fresh, fruity, sweet, grassy
34	ethyl acetate M	C141786	C4H8O_2_	633.5	147.226	1.0998	fresh, fruity, sweet, grassy
35	3-Hydroxy-2-butanone D	C513860	C_4_H_8_O_2_	715.4	196.24	1.32556	butter, cream
36	3-Hydroxy-2-butanone M	C513860	C_4_H_8_O_2_	722.2	201.644	1.07231	butter, cream
37	Furfural D	C98011	C_5_H_4_O_2_	831.1	307.393	1.33734	sweet, woody, almond, bready
38	Furfural M	C98011	C_5_H_4_O_2_	829	305.077	1.08507	sweet, woody, almond, bready
39	Butyrolactone M	C96480	C_4_H_6_O_2_	918.2	425.469	1.08166	cream, fat, caramel
40	*β*-Pinene M	C127913	C_10_H_16_	977.3	532.626	1.21289	resin, green
41	*β*-Pinene D	C127913	C_10_H_16_	976	529.856	1.29733	resin, green
42	*β*-Pinene P	C127913	C_10_H_16_	975	528.009	1.63287	resin, green
43	Camphene	C79925	C_10_H_16_	952.5	484.605	1.20951	woody, camphor
44	*α*-Pinene M	C80568	C_10_H_16_	932.7	449.65	1.21413	fresh, camphor, sweet, pine wood
45	*α*-Pinene D	C80568	C_10_H_16_	934	451.727	1.67103	fresh, camphor, sweet, pine wood
46	*α*-Pinene P	C80568	C_10_H_16_	934.3	452.246	1.72246	fresh, camphor, sweet, pine wood
47	3-Methyl-2-butenal D	C107868	C_5_H_8_O	786	260.17	1.35635	fruity
48	3-Methyl-2-butenal M	C107868	C_5_H_8_O	779.3	253.422	1.08907	fruity
49	1-penten-3-one	C1629589	C_5_H_8_O	667.2	164.389	1.30212	strong pungent odors
50	2-butanone	C78933	C_4_H_8_O	601.2	132.494	1.23571	fruity, camphor
51	2-Heptanone M	C110430	C_7_H_14_O	885.6	376.157	1.26226	pear, banana, fruity, slight medicinal fragrance
52	3-Heptanone D	C106354	C_7_H_14_O	889.3	381.315	1.62728	fruity, grass, oil
53	Butyrolactone D	C96480	C_4_H_6_O_2_	917.2	423.868	1.29782	cream, fat, caramel
54	Limonene M	C138863	C_10_H_16_	1039.9	651.878	1.21338	lemon, sweet, orange, pine oil
55	Limonene D	C138863	C_10_H_16_	1038.1	648.35	1.29803	lemon, sweet, orange, pine oil
56	Limonene	C138863	C_10_H_16_	1038.5	649.055	1.6614	lemon, sweet, orange, pine oil
57	Limonene P	C138863	C_10_H_16_	1038.5	649.055	1.72667	lemon, sweet, orange, pine oil
58	2-Pentylfuran	C3777693	C_9_H_14_O	996.3	572.413	1.25131	bean, fruity, earthy, green, vegetable
59	3-Carene M	C13466789	C_10_H_16_	997.9	575.175	1.21279	citrus, lemon, woody
60	3-Carene D	C13466789	C_10_H_16_	995.3	570.204	1.30035	citrus, lemon, woody
61	3-Carene P	C13466789	C_10_H_16_	995.5	570.756	1.71712	citrus, lemon, woody
62	1-Octen-3-ol M	C3391864	C_8_H_16_O	985.8	550.111	1.15382	mushroom, lavender, rose, hay
63	3-Heptanone M	C106354	C_7_H_14_O	899.9	396.892	1.19904	fruity, grass, oil
64	(*E, E*)-2,4-decadecenal	C25152845	C_10_H_16_O	1331.6	1552.147	1.4288	cucumber, melon, citrus, pumpkin, nutty
65	(*E*)-2-Nonenal	C18829566	C_9_H_16_O	1147.7	898.066	1.41052	fatty, green, waxy, cucumber, melon
66	2-Ethyl-1-hexanol M	C104767	C_8_H_18_O	1044.2	660.107	1.41773	citrus, fresh floral, greasy
67	2-Ethyl-1-hexanol D	C104767	C_8_H_18_O	1045.6	662.876	1.79912	citrus, fresh floral, greasy
68	2-Nonanone	C821556	C_9_H_18_O	1092.8	762.817	1.4051	fresh, sweet, green, herb

Note: RI: retention index, Rt: retention time, Dt: relative migration time.

## Data Availability

Data are contained within the article.
